# Migration-related detention centers: the challenges of an ecological perspective with a focus on justice

**DOI:** 10.1186/s12914-015-0052-0

**Published:** 2015-06-06

**Authors:** Francesca Esposito, José Ornelas, Caterina Arcidiacono

**Affiliations:** 1 ISPA, University Institute, Rua Jardim do Tabaco, 32, 1137-039 Lisbon, Portugal; 2 Department of Humanities, Federico II University of Naples, Via Porta di Massa, 1, 80133 Naples, Italy

**Keywords:** Migration-related detention, Undocumented migration, Ecological perspective, Justice, Health, Equality, Human rights

## Abstract

**Background:**

In recent years, border control and migration-related detention have become increasingly widespread practices affecting the lives of undocumented migrants, their families, and communities at large. In spite of the concern within academia, few studies have directly witnessed the life and experiences of people confined to migration-related detention centers. In the medical and psychological fields, a considerable body of research has demonstrated the pathogenic nature of detention in terms of mental health, showing an association between length of detention and severity of distress. Nevertheless, it was limited to the assessment of individuals’ clinical consequences, mainly focusing on asylum seekers. There currently exists a need to adopt an ecological perspective from which to study detained migrants’ experiences as context-dependent, and influenced by power inequalities. This paper addresses this gap.

**Discussion:**

Drawing upon advances in community psychology, we illustrate an ecological framework for the study of migration-related detention contexts, and their effects on the lives of detained migrants and all people exposed to them. Making use of existing literature, Kelly’s four principles (interdependence, cycling of resources, adaptation, succession) are analyzed at multiple ecological levels (personal, interpersonal, organizational, communal), highlighting implications for future research in this field. A focus on justice, as a key-dimension of analysis, is also discussed. Wellbeing is acknowledged as a multilevel, dynamic, and value-dependent phenomenon.

**Summary:**

In presenting this alternative framework, the potential for studying migration-related detention through an ecological lens is highlighted, pointing the way for future fields of study. We argue that ecological multilevel analyses, conceptualized in terms of interdependent systems and with a focus on justice, can enhance the comprehension of the dynamics at play in migration-related detention centers, providing an effective tool to address the multi-level challenges of doing research within them. Furthermore, they can contribute to the development of policies and practices concerned with health, equality, and human rights of all people exposed to migration-related detention. Consistent with these assumptions, empirical studies adopting such a framework are strongly encouraged. These studies should use mixed and multi-method culturally situated designs, based on the development of collaborative and empowering relationships with participants. Ethnographic approaches are recommended.

## Background

In recent decades, the challenges concerning undocumented migration and border management have become an increasingly sensitive topic at both a European and international level [1]. Although living without a regular status is generally not a choice, but rather a result of limited migration options and procedural barriers within policies of state control [2, 3], in receiving countries, undocumented migrants are often portrayed as “criminals” or “illegitimate others”, unworthy of fundamental rights [4, 5]. The beginning of the ‘war on terror’ following the terrorist attacks of September 11, along with the spread of the global economic crisis, has only served to exacerbate this situation [6–8]. As a consequence, migration has become further securitized, with serious consequences for the lives of all migrants, especially undocumented ones. Studies conducted in various countries have demonstrated the relationship between “undocumentedness” and high health risks for migrants and their families, emphasizing the disparities that exist in terms of access to healthcare, education, work, social services, as well as legal and political rights [9–11].

In this security climate, migration-related detention (M-RD) has become a state mechanism deployed to manage and control individuals and mobile populations [8]. M-RD is the practice - typically based on administrative grounds - of detaining irregular migrants as they violated immigration laws and regulations. Due to the lack of clear and homogeneous national frameworks, migrants in detention often face legal uncertainties and may be detained for up to many months, until being identified and deported, or having their claims adjudicated [12]. In many countries, including Australia, the United States, and much of Europe and Asia, asylum seekers and migrants seeking other forms of humanitarian protection can be detained, pending the decision on the recognition of their status [13].

In light of these observations, a cross-disciplinary academic concern has been growing around the phenomenon of M-RD, and its multiple consequences. Nevertheless, mainly due to the difficulty in gaining access to these centers [14–16], existing contributions have often adopted a view from “above”, failing to directly engage with the people who experience daily life within these institutions [17, 18].

In the medical and psychological fields, much of the research on the human costs of MR-D has documented the effects of detention in terms of mental health. These studies, which have focused mainly on asylum seekers, pointed out an association between the experience of M-RD and poor mental health [19–27]. In particular, some of them have highlighted the fact that asylum seekers' psychological distress worsened over time [15, 22, 28, 29], and that the damaging effects of detention persisted after release [22]. In spite of the evidence presented, this research has the limitation of having focused only on the assessment of individual clinical distress. Alternative to this trend, a line of research on everyday life in M-RD centers is being developed within the field of criminology [14, 17, 30].

In this paper we acknowledge that “medicalized vocabularies for talking about the effects of detention can also be individualistic and ignore detainees’ own framings and social and political contexts”, risking, in this way, to undermine their “political agency by placing them in a passive ‘sick role’” (p. 599) [31]. In this light, the purpose of this paper is to present an ecological perspective, based on recent advances in community psychology, in order to study M-RD contexts and the experiences of people exposed to them. Such a perspective is still lacking in the literature. Both the potential and challenges of the proposed framework will be discussed.

## Discussion

### An ecological perspective from community psychology

As argued at the beginning of the 1980s by Sarason [32], psychology has traditionally been dominated by an emphasis on the individual organism, neglecting the cultural, historical, and contextual influences on human functioning. For a long time, this individual-level emphasis has also dominated research in the area of migration [33]. Therefore, scholars have primarily used individual-level factors to explain variations in the health and wellbeing of migrants, underestimating those processes operating at interpersonal, organizational, institutional, and policy levels [34–36].

The emergence of an ecological psychology domain [37–40] has challenged traditional individual-centered approaches, opening up the possibility of “taking environment into account” (p. 265) [41]. Embracing this alternative worldview, some community psychology scholars developed an ecological analogy to apply to community research and action [41–47]. This perspective integrated a multilevel and dynamic conception of the ecological environment with the principle of developing a collaborative and empowering relationship between professionals and local community members [48]. In particular, drawing on concepts developed in field biology, James Kelly [43] articulated four principles, considered to be the core of the ecological perspective in community psychology: *interdependence*, *cycling of resources*, *adaptation*, and *succession* (See Table 1). Throughout the years, this heuristic has been applied to many contexts of research and intervention [41, 47–52].

**Table 1:** Description of Kelly’s Ecological Principles

Interdependence	Inspired by the concept of ecosystem (i.e., the interdependence among living and nonliving elements of a biological community), this principle states that in changing structures and functions within social environments, the ways individuals and groups cope with events also vary, with a corresponding variation in the performance of adaptive and maladaptive roles. Furthermore, since systems can be viewed as a series of interdependent components, changes in one component reverberate throughout the system.
Cycling of Resources	Referring to how energy is created and transferred within biological systems (e.g., the food chain), this principle emphasizes the importance of looking at the developmental history of a social environment in terms of its management of resources (i.e., how resources are defined, created, distributed, used, exchanged, and transformed).
Adaptation	This principle is based on the evidence that the availability of nutrient substances affects the presence of an organism in a given habitat. It focuses on how environments affect individuals and groups through their demands, norms, values, structures, processes, options and constraints. At the same time, it draws attention to the strategies, and their dynamic evolution over time, which individuals and groups put in place to cope with, adapt to, and try to change the environments in which they live.
Succession	Based on the observation of progressive changes occurring in species structure, organic structure, and in the flow of energy distribution and community production within biological communities, this principle introduces a time perspective. Succession emphasizes how social environments are in a continuous and dynamic course of change that alters their ecology over time, and also with respect to the other principles.

In line with other contributions (e.g., [35, 36, 53, 54]), this paper acknowledges the importance of adopting an ecological perspective from which to study migrants’ experiences as embedded in local contexts, and migration as a context-dependent dynamic influenced by power inequities [33, 36, 54]. Building on these premises, and in order to propose an original framework, Kelly’s four principles [43] are applied to the study of M-RD systems. Each principle is examined at the personal, interpersonal, organizational, and communal level. This choice relies on the assumption that wellbeing is a multilevel, as well as dynamic and value-dependent phenomenon [53, 55]. Such a rich picture of wellbeing, which considers its multiple sources located at different ecological levels, enhances the capacity of scholars to produce positive changes. In accord with this framework, a focus on justice as a contextual dimension operating within and across ecological levels [55] is also discussed. Making use of the literature produced within various academic fields (e.g., criminology of mobility, anthropology, sociology, feminist and critical studies, political geography), potential research questions are highlighted for each principle/dimension.

### Interdependence

This principle suggests that persons and settings are coupled, and that social systems consist of a series of interdependent components, so that a change in one component affects the others [50].

At a *personal level*, the principle draws attention to the *effects of being forced in a condition of detention on migrants’ various spheres of life*, such as health, family and social life, education, work, political and religious freedom, *and person-environment interdependences*. In this regard, it suggests, for example, looking at the impact of M-RD in terms of the redefinition of family roles (detainees are suddenly prevented from staying with, and taking care of their families and children); the changes in self-concept and social status (being a detainee, and a “non-citizen”, becomes the only social recognized identity); the loss of social connectedness and resources (detainees are deprived of all their social networks); the increased risk for physical and mental health. Regarding this latter point, it has already been mentioned in this paper how the negative effects of detention on the mental health of asylum seekers have been documented in many countries [15, 19, 56]. In particular, investigation conducted in Australia [13, 20, 23, 24, 26, 29], as well as in UK [15, 25], and in Japan [19] have demonstrated that M-RD causes the mental health of asylum seekers to deteriorate, and that this deterioration - mainly measured in terms of symptoms of post-traumatic stress disorder, anxiety, and depression - is greater the longer the time in detention [15, 28; 29]. Moreover, high rates of suicide (completed and attempted) and self-harm among detainees have been revealed [16, 21, 23, 29, 56, 57]. In particular, Steel et al. [23] highlighted that while none of their adult participants reported persistent suicidal ideation prior to detention, at the time of assessment, almost all (93 %) had experienced persistent thoughts of suicide, and a third had harmed themselves. Consistent with these results, Sobhanian et al. [21] found a significant reduction in self-harm and suicidal ideation after refugees had been released. Some scholars have emphasized the political nature of these acts, not merely reducible to an expression of desperation [31].

Another critical area of inquiry concerns the *impact of mandatory deportation on the lives of migrants and their families*. For migrants, deportation involves the possibility of returning to countries about which many have little knowledge (because they grew up and lived for many years outside of the country), and where they may not have resources, or even know the language. Some detainees can be deported to places from which they fled due to extreme poverty, or threats to their lives. Recent contributions have pointed out how the impact of the threat and experience of detention and deportation activities not only affect undocumented migrants, but also their family members, the immigrant communities, and the communities at large [58–61]. In particular, children are described as the ones most affected, suffering the consequences of their parents’ distress, the economic hardship faced by their families, and also the stress of abandonment: as a consequence they usually express signs of anxiety, depression, and fear, behavioral problems, and a decline in school performance [60, 61].

Although undocumented detained migrants are the most powerless and vulnerable group within M-RD centers, research should not be exclusively focused on them. Since mental health and wellbeing are nested and sustained by the interconnections with others in specific places, the analysis of how different people, who share the same context, frame and make sense of their particular experience is of central relevance [44]. From this viewpoint, research should provide an understanding of *how various professionals experience M-RD work environments*, and *how these experiences affect their life in terms of person-environment interdependences*. At this level, central issues include how professionals view M-RD centers and make sense of their role within them; the complexities, strengths, and weaknesses they perceive in performing their activities; how they experience power and powerlessness; and how other spheres of their life are affected by their work environment and conditions. For example, when describing the Italian context, Di Martino, Biondi Dal Monte, Boiano, and Raffaeli [62] reported how staff members perceived the centers as detention institutions, often suffering from depressive syndromes and work-related stress.

At the *interpersonal level*, the interdependencies among the various groups that share the same social environment constitute the main focus of inquiry [45]. When focusing on M-RD centers, these *interdependences concern different groups of detainees* (e.g., women and men; migrants of different nationalities and religions; migrants with and without prison experience); *different groups of professionals* (e.g., center staff and immigration officers, non-governmental organization [NGO] practitioners, and faith-based volunteers); *different groups of detainees and professionals*. In order to gain knowledge at this level, the *psychological sense of community*, a construct developed within the field of community psychology, may be of particular relevance. Originally posited by Sarason [63], the psychological sense of community has been defined as the feeling of belonging, mutual influence, fulfillment of needs, and shared emotional connection with other members of one’s group [64]. Although initially applied almost exclusively to territorial communities, the psychological sense of community has more recently been explored in relational communities (e.g., students at school and employees in the workplace), including communities of identity, such as Colored South Africans who have migrated to Australia [65]. Additionally, considering that "individuals have multiple identities and multiple roles, and these identities and roles connect them to multiple communities" (p. 162) [66], the simultaneous existence, operation, and maintenance of multiple psychological senses of community for individuals, both in reference to territorially distinct communities and to sub-communities nested within macro-communities has been highlighted [66].

Envisaging the application of this construct to the study of M-RD centers, some preliminary considerations can be drawn from the work of Bosworth [17]. In her analysis of British detention centers, Bosworth described how the tension between membership based on the sharing of a national identity and sense of belonging to the larger community of detainees was a process constantly in play among detained undocumented migrants. As far as the interdependences between detainees and professionals sharing the same M-RD center are concerned, Bosworth and Bradford [67] stressed how the relationship between the experiences of these two groups is an aspect of the literature that is largely overlooked. Being considered as “non-citizens”, expelled from the communities where they used to (and wanted to) live, detainees are likely to experience a “psychological sense of apartness” (p. 166) [63], isomorphic to their physical apartness. Somehow, feelings of apartness and alienation may also be experienced by the professionals who, in spite of being in a position of greater power, spend a lot of time in these environments of human segregation. Significant in this regard, the extract of an interview with a Greek officer reported by Bosworth et al. [6]: “*It’s much worse than a prison.* Alex told us. *It’s the trap of temporary detention that doesn’t allow us to have the privileges of a prison. They are psychologically distressed here, I would go crazy myself”* (p. 10).

At the *organizational level*, interdependence draws attention to *how different entities* (e.g., immigration office and inter-force police units, managing bodies, and NGOs), *and services* (e.g., medical, psychological, and social), *operating within M-RD centers, may interact among them,* and *how each one is likely to influence the others*. For example, Favel and Silove [57] emphasized the ethical challenges faced by Australian medical staff in balancing the responsibility for providing care to undocumented detained migrants, and the need to respond to the requests from Australian immigration authorities to certify asylum seekers as fit to be detained or be deported.

At this ecological level, another interesting field of study concerns the *interdependences between M-RD centers and external services*. Drawing on interviews with staff and detainees in both prisons and M-RD centers, Bosworth [68] highlighted the growing interdependence between the UK border agency and the prison service. Similar reflections have been developed with respect to the US context [69]. On the basis of this evidence, future studies should delve deeper into the relationships and mutual influences between M-RD and prisons, or other services operating within the community (e.g., healthcare and social services).

At the *communal level*, the focus is on the *interrelations between social, political, economic trends and M-RD systems*. In this respect, a major concern regards the many ways in which policies are filtered and implemented by particular institutions [52]. As pointed out by Di Martino et al. [62], with respect to the Italian situation, since the immigration law has come into force in 1999 no common regulations have been adopted, therefore each M-RD center continues to be framed within local regulations, often not transparent, which may differ and be modified according to changing needs. Scenarios of this type may be common to other national contexts, and scholars should therefore acknowledge these factors in their research.

### Cycling of resources

The principle of *cycling of resources* stipulates that systems may be understood in terms of how they define, harness, create, transfer, and distribute resources [43]. Resources should be broadly conceptualized as including (but not limited to) money, services, infrastructures, and materials, as well as information, competencies, time, and social support. The access to resources is closely related to the power available to individuals and groups.

On a *personal level*, the assessment concerns all the *personal and social resources that may facilitate the detainees’ task of surviving in these environments, thus furthering their resiliencies*. These resources include problem-solving and social skills, as well as social support and positive social identification [55]. For example, McGregor [70] highlighted how faith may help detainees through distressing periods - as a source of energy, hope, and strength, it may foster the resilience of the detainees, turning M-RD centers into spaces of religious revival.

On the side of professionals, *personal and social resources that facilitate the adaptation to M-RD work environments, and the performance of their role* should be examined. In this respect, Bosworth [68] described how the officers that she interviewed, lacking in formal disciplinary powers with respect to prison staff, used to rely on their interpersonal skills in order to “manage” detainees. Furthermore, in her ethnographic study of a British detention center, Hall [8, 71] illustrated how the emergence of shared capacities such as empathy and embodied vulnerability made it possible to challenge and transcend the boundaries between officers and detainees, citizens and others, inside and outside, and served to shape life inside M-RD centers.

On an *interpersonal level*, resource assessment concerns the *characteristics of existing social networks, in terms of their nature and extent* (including formal and informal ties), *the quality of their relationships, and the types of support exchanged* (enacted/perceived support). These dimensions may be considered both with regard to detained migrants and the professionals involved. With respect to detained migrants, one of the few studies addressing their quality of life in detention has highlighted how detainees who felt that they had good relationships - both with officers and other detainees - found the experience of detention less hard to deal with [14, 30]. Future studies may further expand this evidence, taking into account the aforementioned dimensions. For example, the feeling of having good relationships is likely to be associated with the perception of the social support provided within them, which in turn may have a role in buffering the negative effects of detention [72, 73]. To serve as a resource for survival in conditions of hardship, suffering, and injustice, social support must be available in the context (enacted), as well as perceived and mobilized by individuals: exploring these aspects in their mutual interrelations and combined effects in terms of the wellbeing and vulnerability of detained migrants is a challenge to be undertaken in future research.

At the *organizational level*, the array of services (e.g., medical, social, psychological, and legal advice) provided within M-RD centers constitutes an important set of resources to be assessed. In particular, the assessment should concern *services’ accessibility, acceptability, and perceived utility*, in order to understand their *effectiveness in addressing the needs of detainees*. For example, in some countries, such as Italy [62], it has been highlighted how the task of supplying medical services within M-RD centers is taken on by managing bodies that operate independently of local public health agencies. This brings with it the possibility that standards of healthcare and assistance may vary considerably between different centers. Furthermore, in addition to formal services, in M-RD centers there are a range of other settings that are potential sources of support. These include common spaces such as libraries, soccer pitches or other sport fields, and multi-faith prayer rooms (the role of faith in cementing the resilience of the detainees has already been stressed [70]). Future research should consider the *role played by these alternative settings*, shedding light on the *types of support that may be exchanged within them*.

At the *communal level*, the principle of cycling focuses on the *role that local, national, and supranational policies*, *as well as other forces* (e.g., economic trends), *play in defining how resources are created, managed,* and *distributed among and within M-RD centers*. Bacon [74] provided an example of such an analysis, by describing how the privatization of M-RD centers affected the evolution of the detention regime in the UK.

### Adaptation

The principle of *adaptation* concerns the person-environment fit. In particular, this principle suggests the necessity to assess both the qualities of social environments and the challenges that are crucial for individuals and groups (e.g., varying in terms of gender, race, and social status) to survive within them [45].

On a *personal level*, the principle of adaptation focuses on the *strategies that detainees put in place to survive and resist in M-RD centers*, managing to negotiate power and resources. An interesting topic to explore at this level is *the political agency of detainees*. In spite of a wider tendency to conceptualize undocumented migrants as de-politicized subjects [75], some scholars have provided interesting analyses of the forms of resistance put in place by them. For example, Alberti [75] described how, refusing to accept separation from their partners, migrant women detained in Pagani center in Mytilene (Lesvos, Greece), turned down the offer by the Greek government to relocate them and their children to a more “human camp”. Alberti interpreted this collective act of refusing “gentler detention” (p. 141) [75] as a way for women in Pagani to resist the attempt to neutralize their political agency, challenging the representation of detained women as mere victims. Bailey [76] and Grewcock [77] have also provided accounts of the various forms of resistance enacted by detainees in their analysis of some Australian M-RD centers. These included individual and group escapes, hunger strikes, and strikes performed by detainees employed within the centers, self-harm (e.g., lip-sewing), and physical confrontation with staff. Furthermore, McGregor [31] provided an illustration of the controversial dynamics that animated a wide-scale hunger strike organized by Zimbabwean detainees in some British detention centers. In particular, if on one side the scholar stressed the political agency of detainees taking part in the strike, on the other she highlighted the “desperation, distress and divisions” (p. 608) [31] characterizing the protest. Drawing on these considerations, McGregor called for further research assessing the political impact of detainees’ protests.

On the side of professionals, the *strategies put in place to adjust to M-RD work environments and perform their activity* should be taken into account. Examples of these strategies have been illustrated in various contributions [6, 8, 17]. For example, Bosworth [17] reported how, in order to manage the “‘hyper-diversity’ of detention population” (p. 133) [17], professionals tended to generalize and differentiate between nationalities (e.g., Chinese, Nigerians, Jamaicans). In spite of the fact that the strategies adopted by professionals may vary from context to context, and from person to person, they seem to be often drawn on personal initiatives and resources.

Finally, on a personal level, the principle of adaptation suggests the importance of distinguishing between the circumstances faced by different groups of people. Consistent with this assumption, we emphasize the need to take into account the *diversity of experiences, conditions, and needs that characterize migrants in detention.* Children; unaccompanied elderly persons; pregnant women; persons facing health problems, disabilities, or mental health challenges; victims of torture, rape or any other form of violence are part of the population of migrants held in these centers. Therefore, their specific needs should be recognized and addressed. For example, describing children in detention, many scholars have stressed their condition of vulnerability, and the specific risks they face in terms of marginalization, physical and mental harm, developmental and behavioral problems, and possibly abuse [23, 24, 29, 78]. As researchers and professionals involved in the promotion of health and human rights, we have the ethical responsibility to “give voice” to all people - especially those whose voices emerge less - valuing the uniqueness of their stories/experiences, and combating the depersonalization that characterizes these “border zones” (p.14) [8].

At the *interpersonal level*, adaptation may be explored in terms of *competition between members of different groups within a same M-RD center*. This competition is aimed at increasing the access to power and available resources. With respect to detainees, the competition may take place, for example, between groups of different nationalities and religions, as well as between migrants with and without prison experience. In particular, Bosworth [17] highlighted how national identity is one of the most important means by which migrants manage their experience in detention. Therefore, while membership is usually created with co-nationals, the fights with detainees of other nationalities are frequent. Competition may also relate to groups of professionals involved in the everyday life of the centers, especially those belonging to different entities (e.g., center staff, immigration officers, NGOs’ practitioners). Furthermore, competition may concern detainees and professionals. In this respect, Hall [8] described how the social life in the M-RD center that she studied was characterized by divisive and antagonist relations between detainees and officers. Finally, competition may involve groups inside and outside detention facilities. Numerous scholars have provided accounts of confrontation between activists and migration authorities, describing the experiences of “no border” protest camps and the ways in which their antagonist politics took form [75, 79].

At the *organizational level*, the principle of adaptation highlights how each M-RD center has its own *norms, values, beliefs, processes, formal and informal power structures that,* providing specific constraints and challenges, *influence the everyday experiences of people (*i.e.*, detainees and professionals) in these contexts* [41]. To gain insight into these dimensions, the design of the detention facilities, and their geographical location in the territory (e.g., in remote and deserted areas), are crucial sources of information. Details such as the presence of barbed wire, metal detectors, security doors, CCTV cameras, barred windows, isolation cells, and austere décor are usually the embodiment of particular philosophies of security (for an analysis of how the design of prisons and jails is critically related to the philosophy of such institutions, and to society’s approaches for dealing with prisoners, see Wener [80]). Furthermore, they play a critical role in shaping the reactions of detainees and professionals [70].

Another variable to consider at this level is the *degree of sensitivity to cultural diversity*. The degree of cultural sensitivity shown by each M-RD center, also related to the *cultural competence of the professionals* operating within them, is likely to influence migrants’ quality of life in detention, contributing to the exacerbation (in the case of low levels), or the attenuation (in the case of high levels) of migrants’ distress. In light of these considerations, future studies assessing these variables, and their effects, are needed.

At the *communal level*, the *influences of cultural, social, political, and economic factors* are a focus of analysis. In the case of M-RD, these factors include *social norms and beliefs regarding the phenomena of undocumented migration and M-RD, their causes, consequences, and possible solutions*, as well as *immigration policies at local, national, supranational level and broad economic trends*. As highlighted by Toro et al. [52], these factors not only shape the experiences of people - in our case undocumented migrants both held in detention and living in the community - but also affect the institutional responses that are provided for them. For example, Hacker et al. [7] highlighted how, in the US, the approval of the Illegal Immigration Reform and Immigrant Responsibility Act of 1996, and the creation of the Immigration and Customs Enforcement agency after the attacks of September 11, led to an increase in immigration enforcement activity. As a result of this policy change, an increased fear of deportation and distrust in community institutions was observed among documented and undocumented migrants living in Everett (Massachusetts), with serious implications for their health, healthcare access, and effective integration.

Another crucial aspect to be taken into account is the *influence of public opinion*. Becerra et al. [4] pointed out how the perception, deeply rooted in US public opinion, that undocumented migrants are responsible for higher crime rates, and are a cost to taxpayers, has shaped political behavior, leading to the creation of policies and practices lacking rigorous empirical evidence. Focusing on the European context, Bosworth et al. [6] described how, with the onset of the economic crisis in 2008, the attitudes of Greek society towards undocumented migrants dramatically changed. In this climate, politicians used border control and M-RD as political tools to appease public opinion, turning Athens city center into the hub of police operations, which led to the growth of the detention population, and to the construction of larger detention facilities.

A final area of inquiry concerns the *role of media* in influencing the views and fears about undocumented migrants, and, as a consequence, the social and political responses designed for them. Malloch and Stanley [81] highlighted how public concerns in the UK have been heightened by media coverage that portrayed asylum seekers as a problematic, homogenous group that poses material and security threats to British citizens. These authors pointed out how such depictions have also influenced the development of asylum legislations, policies, and practices aimed at achieving tighter border security and internal control, including M-RD. Further research addressing these issues in various local, national, and supra-national contexts is strongly encouraged.

### Succession

The principle of succession defines a time perspective, emphasizing how social environments are not static systems, but in a state of continuous and dynamic change [43]. It stresses the importance of developing a historical and contextual view of the phenomena under study [52].

On a *personal level*, succession invites researchers to develop a *longitudinal understanding of the experiences of people subject to M-RD*. In doing so, they need to address relevant questions such as why people migrate and what their expectations are; how they manage to reach destination countries and through which trajectories; what their settlement experiences are; how their status of documented/undocumented changes over time, and how this influences various spheres of their life; how they end up in M-RD centers; and how is life inside them. Embracing this perspective, Black, Collyer, Skeldon, and Waddington [82] interviewed undocumented migrants held in UK detention centers, describing the diversity of paths that lead to an irregular status. The authors highlighted the perceived safety, the availability of work, and the presence of family and other contacts as key factors in detainees’ decision to migrate to the UK. Pre-migration expectations and motivations, and the complex decision making processes of potential migrants have also been the focus of a study developed by Sladkova at Copán Ruinas (Honduras) [83]. Exploring data across and within individual and community domains, Sladkova highlighted how migration narratives coming from the media, prior migrants, tourists, and coyotes compete for the attention of potential migrants, both encouraging and discouraging Hondurans to irregularly migrate to the US. Based on these results, Sladkova calls for further research on the nature and working of the processes underlying the decision of migrants to emigrate, as well as an examination of how these processes may affect post-migration experiences.

Another area of concern at the personal level, relates to the *long-term effects of detention*. Assessing, at two different moments, the mental health of asylum seekers detained in New York, New Jersey, and Pennsylvania, Keller and colleagues [15] reported significant differences between detainees who had been released, and those who remained in detention. In particular, while the first showed marked reductions in symptoms of anxiety, depression, and post-traumatic stress disorder, the latter were more distressed at follow-up than at baseline. In addition to the evidence presented, this study highlighted some methodological concerns that may arise in conducting longitudinal studies within M-RD centers. These include, for example, the difficulty of conducting repeated interviews with detainees, even when the time interval is relatively short. This difficulty, also reported in other studies [82], is mainly related to the high turnover of detainees due to their transfer to other detention facilities, deportation, or release.

To develop a comprehensive view of the paths of migrants subject to M-RD, succession also suggests looking into the future, namely *post-detention life*. From this viewpoint, accounts of *post-deportation experiences* are particularly meaningful. Studies providing examples of these accounts include that of Ratia and Notermans [84], who reported the experiences of Nigerian women deported from Europe to Nigeria, and Schuster and Majidi [85], who analyzed the possible outcomes of deporting Afghan people. These contributions showed the harmful consequences of deportation activities on people’s lives, and their uselessness as a means of deterring further undocumented migration.

In addition to focusing on detainees, longitudinal studies should also look at professionals, assessing the *long-term effects of working in M-RD contexts*. In this regard, studies developed in correctional institutions have long reported high levels of work-related stress and burnout among correctional officers (for a literature review on this topic, see Schaufeli and Peeters [86]), highlighting the length of experience as a critical factor [87]. Recently, it has also been pointed out that occupational burnout can affect other professionals working in correctional settings, such as correctional psychologists [88]. This evidence invites scholars to extend the assessment to all professionals working in M-RD centers, and not just security officers.

On an *interpersonal level*, the principle of succession draws attention to *how, over time, internal shifts* (e.g., in membership, alliances, culture), *and external forces* (e.g., political, social, and economic changes) *influence the availability and distribution of power and resources within M-RD centers*, as well as *the relationship between different groups*. Contributions embracing this perspective can be found in the literature on prison systems. For example, Crewe [89] described the transformation of staff-prisoner relationships in relation to the transformation of penal power, focusing on the concept of "soft power", and its implications at various levels. Furthermore, by conducting a repeated study (time interval of 12 years) in a UK maximum-security prison, Liebling and Harnold [90] illustrated the changes in the structure and nature of social relationships (staff-prisoner; prisoner- prisoner; staff-staff), and in the role of faith identities in prisoner dynamics. Through their analysis, the authors highlighted how “changes in the structure, culture and values of larger society are powerfully reflected in social relationships and experiences in prison” (p.423) [90].

At the *organizational level*, the focus is on the *assumptions underlying the creation of M-RD centers, and their evolution over time*. In particular, scholars should analyze the historical development of the phenomenon of undocumented migration and how it is framed, as well as the mechanisms implemented by States to deal with it. This knowledge allows a deeper understanding of the current meanings and social functions of M-RD centers, that work as “arts” of national border governance regimes [8]. Moreover, it provides the basis for making predictions about their future course. Hall [8] provided an example of such an analysis. In her ethnographic study of the Locksdon center (UK), she illustrated how the assumptions underlying the everyday operation of the center - based on the intertwinement between control of mobility and production/protection of security - were expressed in daily life, and embodied and achieved through officers’ decisions and actions. The *evolution of institutional cultures and practices within M-RD centers* is another crucial topic to investigate at this level.

At the *communal level*, research should focus on the *ways in which social, economic, legislative, and political changes occur over time*, and on *how they influence the lives of undocumented migrants and the ecology of M-RD systems*. Relevant questions include how changes in migration flows/routes and the promulgation of new regulations are intertwined, and how they influence the everyday practices of immigration enforcement systems, and the experiences of migrants both in and outside detention centers. In a study based in Italy, Di Martino et al. [62] described the developments, since the 1980s, of the framework concerning irregular migration, highlighting how this has been shaped by the periodic existence of exceptional flows of third country nationals configured as “emergency” (e.g., Albanians in 1990s, and North Africans in 2002 and 2011). Shifting the focus to the US context, authors such as Thronson [61] and Sarabia [91] illustrated how immigration laws and policies developed over time, resulting in a growth of the undocumented population, and in the wide spread of “mixed status” families (i.e., families in which all members do not share the same immigration status or citizenship) seriously affected by the tightening of immigration enforcement. Aiming to describe the effects of border control and legalization policies on undocumented migrants’ lives, Saraiba spoke about a process of “perpetual illegalization of migrants” (p. 57) [91] that nullify any attempt of migrants to adjust their status. Although contributions addressing succession at this level are more common, further studies are encouraged, especially if focused on the effects of macrolevel changes on daily life in M-RD.

### A focus on justice

In recent times, in the field of community psychology, Prilleltensky [55] has claimed the role that distinct conditions of justice play in wellness outcomes. In the author’ s view, justice is fundamentally about the “fair and equitable distribution of resources, and about the fair and equitable treatment of other human beings” (p. 9) [55]. In particular, he emphasized the role of distributive and procedural justice, concerned, respectively, with the *what* and the *how*. Both these types of justice - regarding objective as much as subjective resources and goods - act at multiple levels influencing human wellbeing. Various subtypes of justice, specific to each ecological level, derive from these two main types [55]. It will now be illustrated what they are and how they can be applied to the study of M-RD centers, highlighting important research questions.

At the *personal level*, while distributive justice is concerned with what each person gives to herself (e.g., in terms of value, love, and affection), procedural justice has to do with how each person treats herself. In order to apply this rationale to the study of M-RD centers, scholars should taken into account *detainees’ self-conception, self-consideration, and self-esteem* (distributive justice): the analysis of these variables can also reveal *whether detainees have -or not - internalized the self-deprecating views about themselves as “illegal migrants”* with which receiving societies are imbued [4, 5]. Furthermore, the adoption *of behavior causing self-pleasure or,* on the contrary, *self-suffering* should be considered (procedural justice). As previously mentioned, in M-RD centers, acts of self-injury and suicide (attempted and completed) are quite common [16, 29, 56, 57], and they often represent a specific form of protest [31]. Scholars should therefore pay special attention to an examination of these dimensions.

At an *interpersonal level*, while distributive justice refers to the “sharing of goods and responsibilities” (e.g., money and chores), procedural justice is related to the “decision-making process leading to distributions” (p. 8) [55]. In addition, at this level, Prilleltensky argued the role of relational and developmental justice: the former concerning the way of treating others with dignity, fairness and respect, and the latter the expectations about other people’s behavior, and how much these expectations are consistent with their maturational stage. The abuse of power based on a condition of superiority (e.g., physical, psychological, or economic) is a secondary form of developmental injustice pointed out by Prilleltensky.

A consideration of M-RD centers involves focusing on the *distribution of power and resources between the group of professionals and that of detainees, as well as within both groups* (distributive justice). Moreover, it includes the analysis of the *criteria and processes that guide such distribution* (procedural justice).

Relational justice, at this level, is a crucial dimension to look for, and one that should be explored in all relationships (professionals-detainees; detainees-detainees; professionals-professionals): in previous studies, *fairness, humanity, decency*, *and respect at relational level* have indeed been pointed out as critical variables to buffer the harmful effects of detention [30].

Finally, at this level, scholars should assess the *adequacy of the expectations* (e.g., of center staff, and security and immigration officers) *about detainees’ behavior with respect to their maturational stage*, a variable that is linked to the fairness of the treatment that detainees’ receive (see relational justice, organizational level). For example, as already highlighted, children in detention have special needs, face specific problems, and exhibit specific stress reactions [23, 24, 29, 78]. Separation anxiety, depression, nocturnal enuresis, sleep problems, poor appetite, and somatic complaints are some of the challenges faced by children in detention. Thus, particular attention should be given to what is expected from them, by professionals as well as by other detainees. The same rationale may be applied to other groups of detainees such as the elderly, people with disability or those facing mental health challenges. Expecting these people to assume roles or behaviors they are not ready for, or of which they are no longer/not capable, is a form of developmental injustice. Furthermore, *situations of power abuse* (primarily professionals vs. detainees, but also detainees vs. detainees, and professionals vs. professionals) should be a focus of investigation. In such cases it is important to point out if there was *use of violence.*

At the *organizational level*, apart from distributive, procedural, and relational justice, Prilleltensky asserted the role of informational justice, which concerns “the transparency of decision making processes, and the flow of communication” (p. 8) [55]. By focusing on M-RD, a first aspect to consider concerns the *distribution of power, resources, and services between and within different centers* (distributive justice)*.* This can vary greatly, depending also on the various local, national, and supranational policies, and other macro-trends, such as economic ones (see cycling of resources, communal level). However, the *criteria and processes that guide such distribution* (procedural justice) should be subject to detailed analysis.

Relational justice, at this level, is mainly concerned with the *fairness of treatment received by detainees inside M-RD centers*. This dimension is critical for predicting the quality of life in these contexts, and the effects of detention in terms of individual suffering. Furthermore, given our context of study, relational justice is closely linked to cultural justice (see communal level): since M-RD centers are sites of confinement and forced coexistence of different ethnic-cultural groups, the *degree of cultural sensitivity* existing in these institutions, as well as the *cultural competence of the professionals* operating within them, are key-factors to assess (see also adaptation, organizational level). In this regard, it is important to highlight the *occurrence of episodes of discrimination* based on race, ethnicity, or religious beliefs.

Finally, the analysis of informational justice involves the assessment of the *comprehensiveness, transparency, and clarity of information provided to detained migrants about their immigration and asylum cases, as well as about the rules that govern everyday life in detention*. Furthermore, it draws attention to the *directionality of the information flow, and the adequacy of the information exchange between different institutional actors*, whether pertaining to the same body or not (e.g., immigration office, inter-force police unit, managing body, NGOs).

At the *communal level*, four types of justice have been highlighted: distributive, procedural, retributive, and cultural justice. Distributive justice has to do with the distribution, at community and social level, of economic resources, services, and social goods (e.g., safety, education, and health). Procedural justice concerns the fairness of the treatment that people receive within social systems. Aligned with procedural justice, retributive justice relates to how punishment is conceived and to which behaviors it concerns. Finally, cultural justice, which corresponds to relational justice at the level of social groups, is about how entire groups, varying in gender, sexual orientation, culture, race and/or ethnicity, and religion treat each other.

As far as the analysis of the different types of communal justice in relation to M-RD is concerned, we argue that the very existence of institutions for detaining undocumented migrants is in itself evidence of injustice on all the mentioned levels. In terms of what concerns distributive justice, it has been demonstrated that *undocumented migrants*, due to their status of “non-citizens”*, are limited in their access to basic rights enjoyed by national citizens*, including the right to healthcare, education, work, and safety [9–12]. The possibility of being detained represents the ultimate deprivation of rights, undermining, above all, *their right to freedom and self-determination as basic human rights*. Scholars invested in this area should take into account this evidence in their work, highlighting the multiple associated consequences.

Procedural and retributive justice can be analyzed by focusing on the *treatment that undocumented migrants undergo in our societies*: the fact of being detained, sometimes for long or even undetermined periods, *just because of their immigration status*, is evidence of injustice at these levels. Additionally, detainees are frequently victims of offences and violations of rights (e.g., persecution, violence, rape, exploitation), but their condition of irregularity prevents them from receiving the adequate protection, as stated by national regulations and international agreements (e.g., the Universal Declaration of Human Rights). Investigation concerning this last point, i.e., *the relationship between immigration status and the guarantee of rights established by national regulations and international agreements*, is a promising field of study.

Finally, cultural justice can be studied by taking into account the *intertwinement between gender, sexuality, race or ethnicity, class and regimes of M-RD,* a topic whose importance has already been stressed [17, 75, 92]. Key issues worthy of investigation include the question of which groups are more exposed to M-RD and deportation, as well as how their specific condition shapes their experiences in detention.

In concluding, it is important to emphasize that all types/subtypes of justice mentioned should be analyzed and discussed in relation to the different national and local contexts of study. Furthermore, for each context, scholars should point out how the structural inequalities created by M-RD go beyond undocumented migrants, affecting their children and other family members, and the communities at large [59].

### Summary

As highlighted throughout the text, M-RD and its human costs is an area of growing concern both globally and within academia. However, the high complexity of these systems makes the task of studying them a conceptual and methodological challenge for scholars. Some key questions are [17, 93]: What are the best ways to grasp the social life within these contexts, while embracing the diversity of perspectives and experiences that exist within them? How may the human costs of M-RD be revealed, focusing the multi-level factors/processes that are critical in terms of health, wellbeing, and human rights? How is it possible to highlight the strategies of control and oppression exerted, as well as the forms of contestation and resistance that are put in place? How may all the voices, especially those that are more silenced, be empowered through research activities?

Bringing these issues to light does not mean having “ready-made” solutions for their complexities in the conduct of research activities. However, we firmly believe that medicalizing the relative sizes of the experience, as much research continues to do, cannot be an answer, since it involves neglecting the notion that psychosocial determinants of health are not equally distributed among people [55]. Social, economic, and political conditions, as well as power dynamics within societies, have a critical role to play in influencing human wellness. Furthermore, people are not “passive spectators” of their own lives, but active agents who struggle to improve their conditions, influencing their environments.

Based on these premises, and taking into account that M-RD centers are contexts where migrants undergo “persisting conditions of injustice” (p.2) [55], we consider the ecological framework illustrated throughout the text as a useful tool to study these systems, addressing the questions raised. The main features of this framework, and the fields of study that emerge from its application, are reported in Table 2. These include: the longitudinal understanding of the experience of people exposed to M-RD centers; the diversity of strategies used to survive and resist in these environments; detainees’ self-conception, self-consideration, and self-esteem; fairness, humanity, and decency in all relationships as well as concerning detainees’ treatment; the distribution of power and resources between/within groups and M-RD centers, and its guiding criteria; the types of resources available for different groups, and the characteristic of detainees’/professionals’ social networks; sense of community and competition experienced inside the centers; the influence of organizational norms, values, beliefs and formal/informal power structure on individual experiences; the degree of cultural sensitivity/competence of professionals; the interdependences among entities/services both inside and outside M-RD centers; services’ characteristics/effectiveness in addressing detainees’ needs, and the role of alternative settings; the quality of information provided to detainees as well as exchanged between institutional actors; the interrelations between social, political, economic factors and M-RD, and their influences in terms of centers’ ecology and resource allocation; the relationships between immigration status and rights’ access/guarantee; the intertwinement between gender, sexuality, race or ethnicity, class and regimes of M-RD.

**Table 2:** Ecological perspective with a focus on justice as applied to M-RD centers

Interdependence	Personal
	∙ Effects of M-RD on detained migrants’ various spheres of life and person-environment interdependence
	∙ The impact of mandatory deportation on the lives of migrants and their families
	∙ Professionals’ experience of M-RD work environments and its effects in terms of other spheres of life/person-environment interdependences
	Interpersonal
	∙ Interdependences among groups (detainees-detainees; professionals-professionals; detainees-professionals): Psychological sense(s) of community
	Organizational
	∙ Interactions and mutual influences among entities/services within M-RD centers
	∙ Interdependences between M-RD centers and external services
	Communal
	∙ Interrelations between social, political, economic trends and M-RD centers (e.g., ways policies are filtered and implemented)
Cycling of Resources	Personal
	∙ Personal/social resources that facilitate detainees’ task of surviving, furthering their resiliencies
	∙ Personal/social resources that facilitate professionals’ adaptation to M-RD work environments, and the performance of their role
	Interpersonal
	∙ Detainees’ and professionals’ social networks: nature and extent, quality of relationships, types of support exchanged
	Organizational
	∙ Accessibility, acceptability, perceived utility of services within M-RD centers: effectiveness in addressing detainees’ needs
	∙ Role of alternative settings and types of support exchanged within them
	Communal
	∙ Role of local, national, supranational policies/other macro trends in defining how resources are created, managed, distributed among/within M-RD centers
Adaptation	Personal
	∙ Strategies put in place by detainees to survive and resist in M-RD centers: Political agency
	∙ Strategies put in place by professionals to adjust to M-RD work environments and perform their activity
	∙ Diversity of experiences, conditions, needs that characterize migrants in detention
	Interpersonal
	∙ Competitions between members of different groups (detainees vs. detainees; professionals vs. professionals; detainees vs. professionals; insiders vs. outsiders)
	Organizational
	∙ Influence of norms, values, beliefs, processes, formal/informal power structures on the experiences of detainees and professionals
	∙ Degree of cultural sensitivity/cultural competence of professionals
	Communal
	∙ Influences of cultural, social, political, economic, factors: Social norms/beliefs regarding undocumented migration and M-RD, their causes, consequences, and possible solutions Immigration policies at local, national, supranational level and broad economic trends Role of public opinion and media
Succession	Personal
	∙ Longitudinal understanding of the experiences of people subject to M-RD (pre-migration expectations/motivations; migratory trajectories; settlement experiences; documented/undocumented status over time; life in detention)
	∙ Long-term effects of detention
	∙ Post-detention/post-deportation experiences
	∙ Long-term effects of working in M-RD centers (professionals’ work-related stress/burnout)
	Interpersonal
	∙ Impact, over time, of internal shifts and external forces on the availability/distribution of power/resources within M-RD centers, and the relationships between groups
	Organizational
	∙ Evolution of the assumptions underlying the creation of M-RD centers (historical development of undocumented migration/the mechanisms implemented to deal with it)
	∙ Evolution of institutional cultures and practices within M-RD centers
	Communal
	∙ Effects, over time, of social, economic, legislative, political changes on the lives of undocumented migrants/the ecology of M-RD centers (e.g., change in migration flows/routes and promulgation of new regulations)
Justice	Personal (Distributive and Procedural Justice)
	∙ Detainees’ self-conception, self-consideration, self-esteem: internalization of the self-deprecating views about themselves as “illegal migrants”
	∙ Occurrence of behaviors causing self-pleasure or self-suffering
	Interpersonal (Distributive, Procedural, Relational, and Developmental Justice)
	∙ Distribution of power and resources between/within groups (professionals-detainees; detainees-detainees; professionals-professionals)
	∙ Criteria and processes guiding the distribution of power and resources between/within groups
	∙ Fairness, humanity, decency, respect at all level of relationships
	∙ Adequacy of the expectations about detainees’ behavior with respect to their maturational stage
	∙ Situations of power abuse (and related use of violence)
	Organizational (Distributive, Procedural, Relational/Cultural, and Informational Justice)
	∙ Distribution of power, resources, services between/within M-RD centers
	∙ Criteria and processes guiding the distribution of power, resources, services between/within M-RD centers
	∙ Fairness of treatment received by detainees inside M-RD centers
	∙ Degree of cultural sensitivity/cultural competence of professionals: occurrence of episodes of discrimination
	∙ Comprehensiveness, transparency, clarity of information provided to detained migrants about their immigration/asylum cases and the rules that govern the life in detention
	∙ Directionality of the information flow/adequacy of the information exchange between institutional actors
	Communal (Distributive, Procedural, Retributive, and Cultural Justice)
	∙ Restriction of undocumented migrants’ access to basic rights enjoyed by national citizens (e.g., healthcare, education, safety), above all self-determination and freedom: multiple consequences
	∙ Treatment of undocumented migrants on the basis of their status (administrative detention)
	∙ Relationship between immigration status and the guarantee of rights established by national regulations/international agreements (e.g., Universal Declaration of Human Rights)
	∙ Intertwinement between gender, sexuality, race or ethnicity, class and regimes of M-RD (which groups are more exposed to M-RD/deportation and how their condition shapes their experiences in detention)

**Fig. 1:**
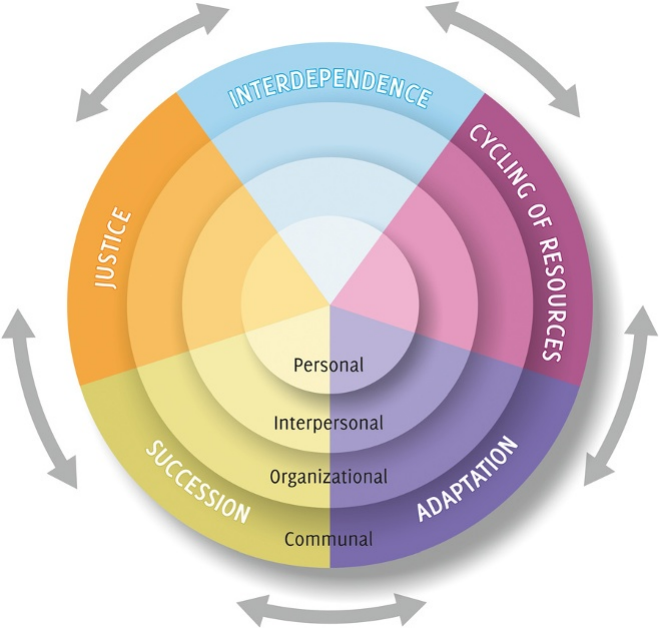
Kelly’s four principles, and the dimension of justice across multiple ecological levels of analysis. Interdependence, Cycling of Resources, Adaptation, Succession, and Justice are interdependent components, whose effects, across multiple ecological levels (personal, interpersonal, organizational, communal), are interactive rather than additive

It is important to stress that *Interdependence*, *Cycling of Resources*, *Adaptation*, *Succession*, and *Justice* are here intended as interdependent components, whose effects are interactive rather than additive. Figure 1 provides a visual representation of the four principles and the dimension of justice as applied to the multiple ecological level of analysis (personal, relational, organizational, communal).

Consistent with this theoretical rationale, at a methodological level we assert the need to design mixed and multi-method culturally situated studies [45, 46], based on the development of collaborative and empowering relationships with participants [47, 48]. Trust in the research relationship is, in this sense, a precondition to create a solid basis for collaborative work [45]. In the literature on M-RD, it has been emphasized how linguistic, cultural, and gender differences, along with the high turnover of detainees, the distrust towards outsiders, and the high levels of suffering and anguish for the future constitute critical challenges for the scholars who wish to build trust relationships with detained migrants [30, 82, 94]. In addition, feelings of distrust towards researchers/outsiders may also be shared among professionals, who are easily susceptible to feeling attacked because of their role in maintaining M-RD centers. In light of these considerations, research adopting an ethnographic approach is recommended. By valuing the ways in which scholars’ identities and experiences, alongside their power and privileges, can influence the research process, ethnographic approaches may be particularly useful for developing meaningful and trustful research relationships [94]. The potential of ethnography for bridging universal questions with situated experiences of individuals and groups has recently been stressed in community psychology [95].

To conclude, in proposing this framework we do not claim its exhaustiveness, nor that it represents the “only right way” to conduct research in M-RD contexts. The existing research on migrant detention is not without value. Each research study is a “story” in itself, requiring decisions about the research design, questions, methods, analytical techniques that greatly depend on the sensitivity of the researcher, and the opportunities for action in the particular contexts she/he aims to study [45]. However, we argue that this framework may enhance our understanding of the dynamics at play in M-RD centers and the multiple associated human costs, providing the basis for planning actions that foster the health, equality, and human rights of all people exposed to these contexts - mainly undocumented migrants. Ultimately, we hope that this paper will facilitate work in this area, giving directions for future studies.

## Abbreviations

M-RD: Migration-related detention
NGOs: Non-governmental organizations
